# Insulin Signaling as a Mechanism Underlying Developmental Plasticity: The Role of *FOXO* in a Nutritional Polyphenism

**DOI:** 10.1371/journal.pone.0034857

**Published:** 2012-04-13

**Authors:** Emilie C. Snell-Rood, Armin P. Moczek

**Affiliations:** 1 Department of Biology, Indiana University, Bloomington, Indiana, United States of America; 2 Department of Ecology, Evolution and Behavior, University of Minnesota, St. Paul, Minnesota, United States of America; The Walter and Eliza Hall of Medical Research, Australia

## Abstract

We investigated whether insulin signaling, known to mediate physiological plasticity in response to changes in nutrition, also facilitates discrete phenotypic responses such as polyphenisms. We test the hypothesis that the gene *FOXO* – which regulates growth arrest under nutrient stress – mediates a nutritional polyphenism in the horned beetle, *Onthophagus nigriventris*. Male beetles in the genus *Onthophagus* vary their mating strategy with body size: large males express horns and fight for access to females while small males invest heavily in genitalia and sneak copulations with females. Given that body size and larval nutrition are linked, we predicted that 1) *FOXO* expression would differentially scale with body size (nutritional status) between males and females, and 2) manipulation of *FOXO* expression would affect the nutritional polyphenism in horns and genitalia. First, we found that *FOXO* expression varied with body size in a tissue- and sex-specific manner, being more highly expressed in the abdominal tissue of large (horned) males, in particular in regions associated with genitalia development. Second, we found that knockdown of *FOXO* through RNA-interference resulted in the growth of relatively larger copulatory organs compared to control-injected individuals and significant, albeit modest, increases in relative horn length. Our results support the hypothesis that *FOXO* expression in the abdominal tissue limits genitalia growth, and provides limited support for the hypothesis that *FOXO* regulates relative horn length through direct suppression of horn growth. Both results support the idea that tissue-specific *FOXO* expression may play a general role in regulating scaling relationships in nutritional polyphenisms by signaling traits to be relatively smaller.

## Introduction

Variation in the quality and quantity of nutrition is a ubiquitous challenge during development both within and across generations. Organisms have evolved a wide range of behavioral, developmental, and physiological mechanisms to cope with such variation. Central among the developmental and physiological mechanisms is the insulin signaling pathway, which permits major adjustments in growth, development, body size, lifespan and behavior in response to nutrition (reviewed in [1]–[4]). In times of plenty, insulin or insulin-like peptides promote, via the insulin receptor, a variety of cellular processes such as cell division, metabolism, and protein synthesis [5]–[9]. During periods of lower food availability, the decrease in insulin or insulin-like peptides results in the activation of the transcription factor forkhead-box-subgroup O, or *FOXO*, which subsequently inhibits growth [10]–[12], and promotes immunity [13], stress resistance [14], lifespan [15]–[17], lipid metabolism [18], and increased insulin sensitivity through up-regulation of the insulin receptor [12], [19].

Insulin signaling is a highly conserved pathway that allows organisms as diverse as mammals, insects and nematodes to cope with fluctuations in diet through proportional changes in growth and life history traits [20]. However, many organisms adopt qualitatively different strategies or grow vastly different traits depending on nutrition. For instance, variation in nutrition results in the adoption of different mating tactics in many species of insects and fish [21], [22] and different reproductive castes in many social insects [23], [24]. In mammals, diet is hypothesized to play a major role in the development of different metabolic syndromes [25]. This raises the questions whether insulin signaling has been co-opted from a primary role in basic physiology to a mechanism of phenotypic plasticity in response to nutritional variation.

Several lines of evidence suggest that insulin signaling should be a common mediator of plastic responses to variable nutritional environments. First, insulin signaling plays a role in determining the relative size of tissues and organs ([11], [12], [26]; reviewed in [27], [28]). Changes in the relative size of tissues across a nutritional gradient are important components of plasticity. Such flexibility and diversity in allometric scaling relationships is thought to be regulated by insulin signaling [27], [29], [30]. For example, shallow-sloped correlations between body size and genitalia [31], in contrast to isometric scaling relationships of many other traits, may be due to lower sensitivity of genitalia to insulin signaling, possibly through changes in insulin receptor density [32]. Additional evidence linking insulin signaling to plasticity comes from gene expression studies. Candidate genes in the insulin signaling pathway show different expression patterns between polyphenic morphs, such as different reproductive morphs of paper wasps [33], reproductive, worker or nurse castes of honey bees [34]–[36], predator-induced morphs of *Daphnia* [37], or sneaker and fighter morphs of horned beetles [29].

We sought to test the role of insulin signaling in nutritionally induced phenotypic plasticity using the sneaker-fighter polyphenism in horned beetles. Beetles in the genus *Onthophagus* construct brood balls (of dung) that support the entire larval development of individual offspring. The body size of an adult is largely influenced by the size and nutritional quality of their brood ball [38], [39]. Emerging adult males adopt distinctly different reproductive tactics depending on their own body size. Large adult males use horns in aggressive contests with other males over females and their tunnels [40]. In contrast, small males grow only horn rudiments and adopt sneaker tactics, digging side tunnels to access to females, or sneaking copulations with females as horned males fight [40]. Small, hornless males are more maneuverable in tunnels [21], and instead of growing horns [41], [42], they often invest in relatively larger genitalia and/or ejaculates [43], [44].

We tested the hypothesis that insulin signaling plays a role in the development of this nutritional polyphenism in mating tactics in horned beetles, using a combination of observation and manipulation of patterns of gene expression. We chose to focus on one important player in the insulin signaling pathway, *FOXO*, because it has been linked to variation in scaling relationships in a range of systems [5], [11], [12], [16], [45] and hypothesized to be an important regulator of horned beetle polyphenisms [29]. Recent microarray analyses [46] provided the first empirical support of this hypothesis by documenting elevated *FOXO* expression levels in developing horn tissue, relative to expression in the abdomen, of small, hornless males compared to large, horned males. Furthermore, recent work in *Drosophila* suggests that *FOXO* expression may regulate tissue-specific responses to nutritional variation [30], an important criterion for a gene involved in a nutritional polyphenism.

We sought to test two hypotheses for the potential role of *FOXO* in this nutritional polyphenism. First, we investigated the hypothesis that *FOXO* regulates horn size relative to body size. Mechanistically, in this case, the activation of *FOXO* in the developing horn tissue of small, nutritionally stressed males is expected to repress horn growth. This hypothesis predicts that *FOXO* expression would be elevated in the developing horn rudiments of small males, and that knockdown of *FOXO* would result in larger horn length for a given body size. Second, we address the hypothesis that *FOXO* regulates genitalia investment relative to body size. Past research has suggested that costly horns [41], [42] may tradeoff with investment in genitalia and ejaculates [43], [44]. Mechanistically, the growth of a large horn in a beetle may trigger *FOXO* expression in other developing regions, such as the genitalia, resulting in the repression of their growth. This hypothesis predicts that *FOXO* expression will be elevated in developing abdominal and genitalia tissues of large, horned males and that knockdown of *FOXO* would result in larger genitalia size for a given body size. Our results suggest that *FOXO* plays an important role in nutritional polyphenisms, negatively regulating relative trait size for both horns and genitalia.

## Results

### *FOXO* Sequencing

We sequenced a 1204 bp region of the *FOXO* transcript (912 bp of coding sequence) in *Onthophagus nigriventris*. Using BLASTp, this region significantly matched *FOXO* from *Tribolium castaneum* (e value = 3e^−116^) with 79% matching amino acid identity. Additionally, we sequenced *FOXO* in several species of *Onthophagus* and aligned the protein sequences. We found that *Onthophagus* beetles, relative to *Tribolium*, have a large insertion (5–9 amino acids) in the coding region of the gene (Fig. S1). For subsequent analyses, we focused on a region of the gene past the conserved forkhead domain (see Fig. S1 and methods for more details).

### *FOXO* Expression in Untreated Individuals

**Figure 1:**
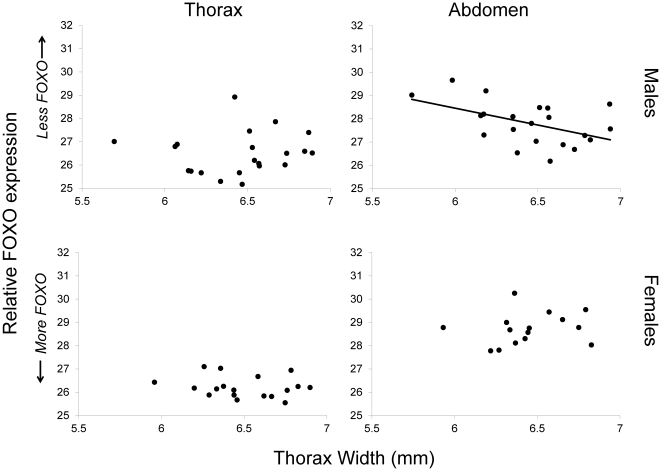
Patterns of *FOXO* gene expression in males and females. Real-time quantitative PCR was used to measure expression of *FOXO* in 21 male and 18 female untreated individuals sacrificed as first-day pupae. Gene expression was measured in both the thoracic and abdominal epidermis. We used ANOVAs to test for effects of body size on *FOXO* expression, relative to the expression of the control gene (actin). Separate ANOVAs were run for each tissue and sex (see Table 1). Shown are leverage plots from an ANOVA that included body size (thorax width) and expression of a control gene (actin Ct) as independent variables (*FOXO* Ct was the dependent variable); thus, relative FOXO expression is plotted as the dependent variable. The body size range included small, hornless males to large, horned males.

Contrary to our expectations, *FOXO* expression, relative to that of a control gene (actin) was not correlated with body size in the developing horn region (the thoracic epidermis) of either males or females sacrificed as first day pupae (Table 1, Fig. 1). However, *FOXO* expression was positively correlated with body size in the abdominal epidermis of untreated males: larger males had consistently and significantly higher relative *FOXO* expression (Table 1, Fig. 1). Because this result was in contrast to our expectation of higher *FOXO* expression in the prothorax of small, hornless males, yet revealed an unexpected elevation of *FOXO* expression in the abdomen of large males, we wished to further investigate location and level of *FOXO* expression in the abdomen of males.

**Table 1:** Expression of *FOXO* varies with body size.

	Thorax		Abdomen	
	Body size	Control gene	Body size	Control gene
Males	*F_1,18_* = 0.18	*F_1,18_* = 6.78* b = 0.71	*F_1,18_* = 5.68*	*F_1,18_* = 0.50 b = 0.13
Females	*F_1,15_* = 0.54	*F_1,15_* = 8.42** b = 0.41	*F_1,12_* = 0.50	*F_1,12_* = 2.93 b = 0.27

Real-time quantitative PCR was used to measure expression of *FOXO* in 21 male and 18 female untreated individuals sacrificed as first-day pupae. Gene expression was measured in both the thoracic and abdominal epidermis. Shown are F values and slope estimates (coefficients of the least squares linear model) from ANOVAs that include effects of both body size (thorax width) and expression of a control gene (actin).

* *P*<0.05.

** *P*<0.01.

**Figure 2:**
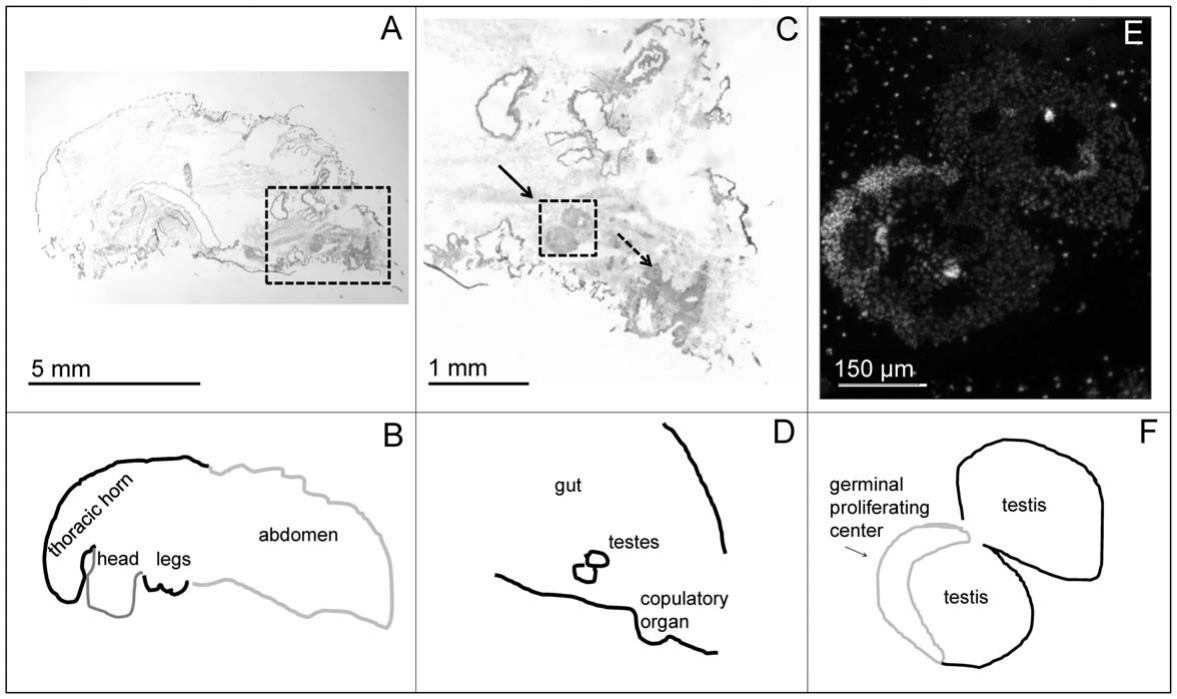
*FOXO* expression in large, horned male. Shown are *in situ* hybridization images of a representative sagittal-medial sections of large, horned males, obtained from *O. nigriventris* pupae during the first day of the pupal stage. A, B) *FOXO* expression in the posterior-ventral region of the abdomen. (C, D) A close-up of *FOXO* expression (dotted rectangle in (A)) in the putative developing genitalia, including the putative testes (solid arrow) and copulatory organ (dashed arrow). This may also include expression in the fat bodies. The coloring of the cuticle is a normal artifact. (E, F) DAPI stain of (C) showing putative testes, including a region of densely packed cells, the putative germinal proliferating center.

**Figure 3:**
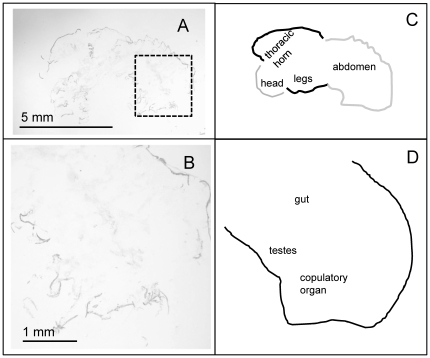
Lack of *FOXO* expression in small, hornless male. Shown (right panels) is a representative *in situ* hybridization showing FOXO expression in small, hornless male pupae *O. nigriventris* (day 1). (B) shows a close-up of the square area highlighted in (A). (C) and (D) label the different body regions shown in the section.

*In situ*-hybridizations were performed on males to investigate spatial patterns of gene expression. In line with the qPCR results reported above, we observed *FOXO* expression in several locations in the abdomen of large, horned males, but not in the comparable location in small, hornless males (observed in three replicates for each morph; Fig. 2, 3). Based on the location and shape of these clusters of expression (2–4 oval clusters in the ventral posterior abdomen, adjacent to a single rectangular structure), we can putatively identify *FOXO* expression in the developing testes and copulatory organs of large, horned males. A DAPI stain of the putative testes region showed a cell-rich area at the anterior end of the oval shaped structure (Figure 2E), consistent with the densely clustered, mitotically dividing cells of the anterior testes (“germinal proliferating center:” [47]). While the region of *FOXO* expression was consistent with expression in the genitalia, we cannot rule out additional expression occurring in the fat body. There was little detectable (if any) *FOXO* expression in the developing horns or prothorax of any of these samples (Fig. 2, 3).

Taken together, our expression results suggest that *FOXO* is expressed in the developing genitalia (and possibly the fat body and/or adjacent areas in the abdomen) of large, horned males, but much less abundant in the corresponding tissue regions in small, hornless males. Contrary to our initial expectations, *FOXO* expression is indistinguishable in developing pupal horn tissues of large and small males.

### *FOXO* RNAi Knockdown

#### RNAi phenotypes

**Figure 4:**
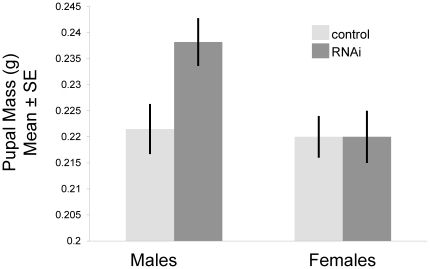
Effect of *FOXO* knock-down on body size. Shown is mean and standard error of pupal mass for beetles were injected with at least 1 ug ds *FOXO* RNA relative to control-injected individuals. There was a significant effect of *FOXO* knockdown on body size in males, but not females (see Table 2).

*FOXO* RNAi had significant effects on body size and development time in males, but not females. Relative to control-injected individuals, RNAi males had significantly larger pupal thorax widths and pupal weights, extended length of their third instar, and marginally significantly larger adult thorax widths (Fig. 4, Table 2). In contrast, none of these variables differed between control-injected and RNAi females. The body size response in males was proportional to the degree of *FOXO* knockdown as approximated by the amount of dsRNA injected (see Table S1). However, the degree of *FOXO* knockdown did not affect relative horn length (*F_1,41_* = 0.58, *P* = 0.45) or genitalia scaling relationships and thus was not included in subsequent analyses.

**Table 2:** Differences in body size and development time between control-injected and treatment individuals.

	Males		Females	
	Mean (SE)	Statistics	Mean (SE)	Statistics
Pupal Thorax Width (mm)	C: 6.17 (0.06)F: 6.32 (0.05)	*F_1,62_* = 3.70*P* = 0.05	C: 6.31 (0.08)F: 6.17 (0.07)	*F_1,57_* = 1.65*P* = 0.20
Pupal Mass (g)	C: 0.22 (0.005)F: 0.24 (0.05)	*F_1,63_* = 6.17*P* = 0.02	C: 0.22 (0.004)F: 0.22 (0.005)	*F_1,57_* = 0.04*P* = 0.83
Length of 3^rd^ Instar (days)	C: 18.7 (0.36)F: 20.0 (0.38)	*F_1,51_* = 6.48*P* = 0.01	C: 18.6 (0.42)F: 18.8 (0.45)	*F_1,52_* = 0.05*P* = 0.82
Adult Thorax Width (mm)	C: 5.89 (0.06)F: 6.03 (0.06)	*F_1,52_* = 2.84*P* = 0.09	C: 5.91 (0.06)F: 5.91 (0.06)	*F_1,44_* = 0.006*P* = 0.93

*FOXO* (F) knockdown individuals were treated with either 1.0, 1.75, or 2.5 ug of dsRNA, while control (C) individuals were injected with 1.0 ug bacterial dsRNA. Shown are results of ANOVAs testing (independently) for effects of treatment (control versus knockdown) on size and development time for males and females.

**Figure 5:**
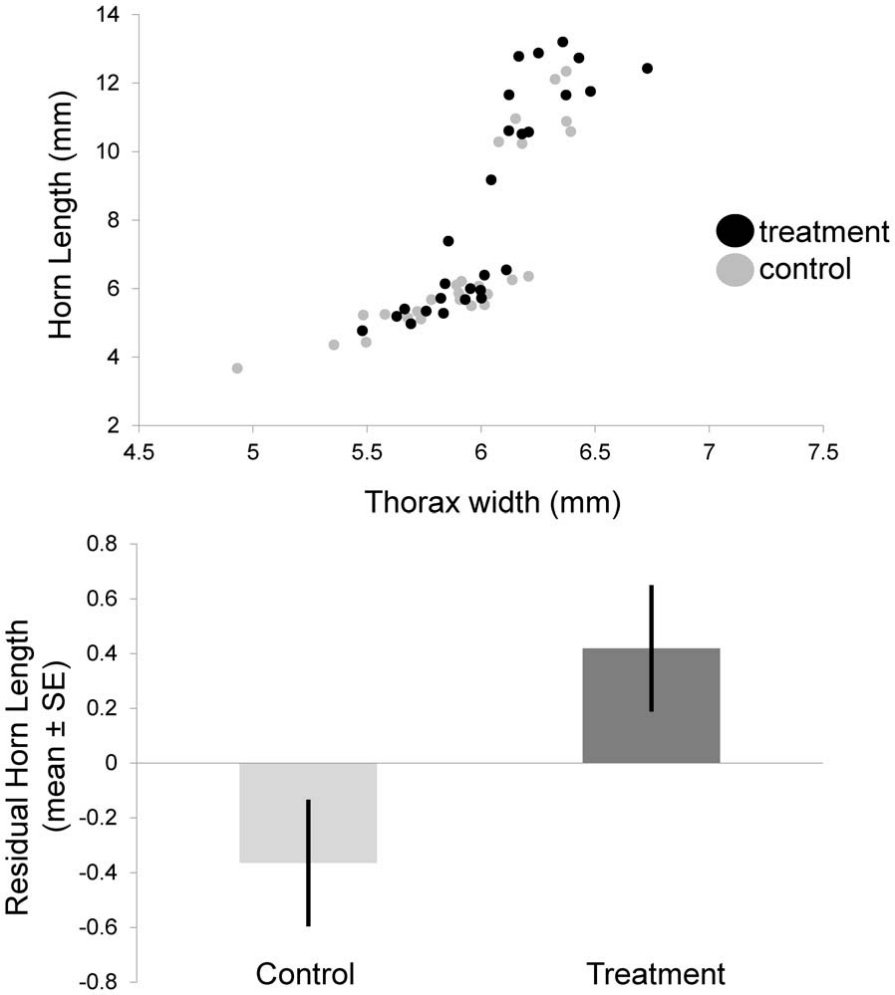
Effect of *FOXO* knockdown on horn-body size scaling. Allometry between body size (thorax width) and thoracic horn length in control-injected and *FOXO* knockdown individuals (treated with 1.0, 1.75, or 2.5 ug dsRNA). The lower panel shows the difference in relative horn length between control-injected and *FOXO* knockdown individuals – the difference was modest, but significant (*P* = 0.02).

We also detected a moderate effect of RNAi on horn expression. Relative to control-injected individuals, RNAi male adults had significantly longer horns relative to their body size (Fig. 5, *F_1,52_* = 5.75, *P* = 0.02). Residual horn length did not differ between control and treatment males in the pupal stage (*F_1,62_* = 1.67, *P* = 0.20). In females, which express a small horn as pupae and a ridge as adults, we observed no differences in horn-body size relationships between control-injected and RNAi individuals for either the pupal (*F_1,57_* = 0.59, *P* = 0.44) or adult stage (*F_1,44_* = 0.28, *P* = 0.59).

**Figure 6:**
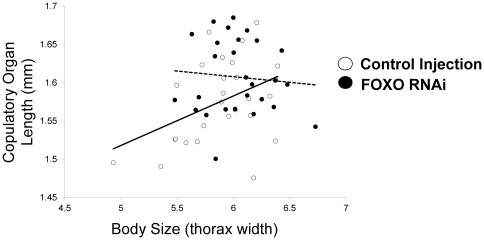
Effect of *FOXO* knockdown on body size-copulatory organ scaling. Allometry between body size (thorax width) and copulatory organ length (paramere) in control-injected and *FOXO* knockdown individuals (treated with 1.0, 1.75, or 2.5 ug dsRNA). The body size range included small, hornless males to large, horned males (see Fig. 5).

*FOXO* knockdown also had significant effects on the relative size of the male copulatory organ. Overall, *FOXO*-knockdown individuals had larger copulatory organs for a given body size relative to control-injected individuals (*F*_1,50_ = 3.93, *P* = 0.05; mean (se), control: 1.58 (0.009), *FOXO*: 1.61 (0.009), see also Fig. 6). There was a marginally significant interaction between body size and treatment (*F*_1,50_ = 3.23, *P* = 0.07; size effect: *F*_1,50_ = 1,28, *P* = 0.26; see also Fig. 6). Combined, these RNAi results suggest that *FOXO* regulates not only body size, but also the *relative* growth of traits important in a nutritional polyphenism – horns and genitalia.

#### Validation

We used quantitative real-time PCR to validate the RNAi knockdown. We harvested thoracic and abdominal tissue from 20 male and female first-day pupae (within 24 hours of pupation) of control-injected (N = 11) and *FOXO*-injected (1.0 ug dsRNA, N = 9) individuals. We tested for differences in relative *FOXO* expression in an ANOVA that included expression of the control gene actin (thorax: *F_1,16_* = 2.33, *P* = 0.15; abdomen: *F_1,14_* = 0.24, *P* = 0.63), and a measure of body size (pupal mass: thorax: *F_1,16_* = 10.4, *P* = 0.005; abdomen: *F_1,14_* = 0.63, *P* = 0.44). We found a significant effect of dsRNA injection on *FOXO* expression (Figure S2, thorax: *F_1,16_* = 5.89, *P* = 0.02; abdomen: *F_1,14_* = 6.83, *P* = 0.02). Using the methods of Pfaffl [48], this corresponded roughly to a 0.80 and 1.08 fold reduction in *FOXO* expression, relative to control-injected individuals, for the abdomen and thorax, respectively. Because our validation was on individuals injected with 1.0 ug *FOXO* dsRNA, all analyses focused on individuals injected with at least 1.0 ug dsRNA.

## Discussion

We present evidence that suggests insulin signaling plays a role in nutrition-induced phenotypic plasticity in horned beetles. We found only modest support for a role of *FOXO* in the direct regulation of relative horn size in males. However, our data provide support for the hypothesis that *FOXO* regulates the relative size of male genitalia, thus playing an important role in trait integration across polyphenic morphs. These results are consistent with recent observations that *FOXO* expression regulates tissue-specific responses to nutritional variation in *Drosophila* [30]. We discuss our findings in the context of what is known about *FOXO* and insulin signaling, and future directions for investigating *FOXO* as a player in nutritional plasticity.

### Conservation of the Insulin Signaling Pathway

Our data are consistent with previous studies on insulin signaling and *FOXO* in particular. We found that *FOXO* knockdown affected both development time and body size (Table 2). Previous studies on *Drosophila* have shown that manipulations of insulin signaling prior to the larval critical weight affect development time, and manipulations that occur after the larval critical weight affect body size [32]. While the critical weight is unknown in this species, our knockdown likely occurred just prior to, and affected animals for several days following, critical weight, at least as defined in *Drosophila* (mass at which 50% of animals will survive to pupation without further feeding, [49]). We injected beetles between day 6 and 10 of the third larval instar, just before peak mass occurs (between day 10 and 12; [50]) and effects of the knockdown were measurable using qPCR in first day pupae. Thus it is likely that we affected development periods both before and following critical weight.

We found that *FOXO* knockdown increased adult body size (Table 2, Fig. 4). Previous empirical work has shown that increased insulin signaling (through an increase in expression of insulin-like peptides or the insulin receptor) causes an increase in body size [5], [7], while decreased insulin signaling causes a decrease in body size [5], [6], [8], [9], [32]. Similarly, a range of studies in *Drosophila* have found that over-activation of the growth inhibitor *FOXO* results in decreased body size [10]–[12]. Thus, we should expect decreased *FOXO* expression to result in an increase in body size, as found in our study (Table 2, Fig. 4). In contrast to this expectation, one previous study in *Drosophila* found that a *FOXO* null mutation had no effect on adult body size [10], though it is possible that the expected increase in body size is dependent upon sufficient nutrition. In support of this idea, we also observed a significant increase in development time in males (Table 2). Because we manipulated *FOXO* prior to the critical weight, it is possible that larger body size could have been achieved through a flexible increase in development time (and resulting total nutrient pool). Variation in nutrient availability or timing of knockdown may result in variation among studies in the effect of *FOXO* knockdown on body size.

We also found that the effect of *FOXO* on body size in the present study was specific to males (Table 2, Fig. 4), recalling previous work that found sex-specific effects of *FOXO* knockdown [15]. It is unclear exactly why *FOXO* knock-down would have sex-specific effects on body size. However, the result recalls the idea that sexually selected traits generally evolve greater degrees of condition dependence [51], resulting in greater degrees of nutritional sensitivity in many traits in males relative to females [52]. Given that body size is under sexual selection in male beetles (due to its link with contest outcome, reviewed in [53]), our results are consistent with this idea.

Our results also suggest a role of *FOXO* in the regulation and establishment of scaling relationships. Previous studies have shown that changes in insulin signaling in specific tissues, including *FOXO* activity, can result in organ-specific changes in size [5], [11], [12], [16], [28], [45]. Such effects are what make the insulin signaling pathway a prime candidate for determining diversity in trait allometries, or differences in shape within and between species and nutritional environments. For example, a tissue-specific change in *FOXO* expression (as suggested in [29]) or insulin receptor density (as suggested in [27]) has the potential to change the relative size of an organ. Indeed, target gene responses to *FOXO* activity vary with tissue type [54], [55]. Consistent with these expectations, and as we discuss in more detail below, we found that *FOXO* expression varied with tissue types (Fig. 1) and knockdown resulted in changes in trait-body size relationships for both thoracic horns (Fig. 5) and genitalia (Fig. 6).

Overall, these data suggest that *FOXO* may partially mediate its effects on allometry through differential expression. While the classical view of *FOXO* action considers changes in activity through protein phosphorylation (rather than differential expression) (reviewed in [2], [56]), recent work suggests a more complex picture [28], [56], [57]. The present research provides further support for the emerging idea that changes in *FOXO* expression may be just as important as changes in *FOXO* activity in generating changes in size and shape [30]. However, future work will be necessary to determine the relative importance of *FOXO* expression versus activation in the development of polyphenisms.

### *FOXO* Expression Is Linked to a Nutritional Polyphenism

Several lines of evidence implicate *FOXO* in the development of the fighter-sneaker polyphenism in horned beetles in the genus *Onthophagus*. First, we found a correlation between *FOXO* expression and body size (Fig. 1) – a result of larval nutritional conditions. This relationship was restricted to males, the sex with pronounced nutritional polyphenism. Specifically, we found that *FOXO* expression in the abdominal epidermis was positively related to body size in males. *FOXO* expression in the developing horn tissue of the thoracic epidermis, however, was not. This result confirmed previous microarray analyses which indicated that *FOXO* expression in the thoracic tissue, *relative* to the abdominal tissue, was higher in small, hornless males than in large, horned males [46]. However, contrary to initial expectations this relative difference was *not* due to higher expression of *FOXO* in the thorax of small compared to large males; instead it was due to higher *FOXO* expression in the *abdomen* of large compared to small males. We sought to clarify the spatial pattern of expression of *FOXO* in the abdomen using in situ hybridization.

In situ hybridizations suggested that *FOXO* expression was most pronounced in the putative developing genitalia (testes and copulatory organ) of horned males (Fig. 2, 3), although it is possible expression was also present in the fat body. This result is consistent with the qPCR result showing increasing *FOXO* expression with body size in the abdomen of males. However, given that the tissue used in the qPCR analyses was dorsal abdominal tissue, while the genitalia are located more ventrally, it suggests that the qPCR results may have been driven by *FOXO* expression in a range of abdominal tissues such as the fat body, genitalia and/or other tissues adjacent to the dissection location (first day pupae have indistinct borders between internal tissues).

Taken together, the results of the qPCR and in situ hybridization run counter to the initial hypothesis that small, nutritionally stressed males would express *FOXO* in the thoracic tissue, resulting in the repression of horn growth [29]. Instead, these observations support the hypothesis that horn growth in large, horned males may trigger nutrient stress and *FOXO* expression in other body regions which signals organs within those tissues to grow proportionally smaller. We sought to test this hypothesis further via knockdown of *FOXO* expression.

We used RNAinterference to test the degree to which *FOXO* affected horn and genitalia size, relative to body size. We found that *FOXO* knockdown had significant effects on genitalia size relative to body size (Fig. 6): specifically, *FOXO* knockdown individuals had relatively larger genitalia relative to control-injected individuals. This observation is consistent with the hypothesis that *FOXO* may regulate relative genitalia size and that *FOXO* expression in the abdomens of large males lowers genitalia investment. We also observed that the slopes of the body size-genitalia relationship were different between the two treatments (although this interaction was only marginally significant) such that relative differences in genitalia size were most pronounced in smaller sized individuals. It is possible that growth of horns in large males prevented genitalia growth by means of nutrient limitation despite the signal for continued growth stimulated through depressed *FOXO* expression, although future research is necessary to test this mechanism. *FOXO* RNAi also significantly affected the size of thoracic horns relative to body size, although the effect was modest overall (Fig. 5). Relative to control-injected individuals, RNAi individuals expressed larger horns for a given body size (Fig. 5), with the effect being most pronounced in several intermediate-sized and large individuals. This is consistent with the initial hypothesis that *FOXO* expression in the developing thoracic epidermis may regulate horn growth. However, given that *FOXO* expression (in first day pupae) was weak in the prothorax of small, hornless males at the first day pupae stage (Fig. 1, 3), this suggests that a corresponding window of nutrition-mediated differential *FOXO* expression must come earlier in development, for instance during the pre-pupal stage when horn tissue is proliferating. Furthermore, the RNAi effect was most pronounced for intermediate and large males, while the original hypothesis predicted the effects to be most pronounced in small, nutritionally stressed males. Regardless, both the horn and genitalia results taken together suggest that *FOXO* may generally regulate relative trait size by suppressing growth.

### Conclusions and Future Directions

In summary, our results suggest that *FOXO* plays a role in the development of the *Onthophagus* polyphenism. Our data provide limited support for the initial hypothesis that *FOXO* regulates relative horn length through suppression of horn growth. However, both expression and knockdown data suggest that *FOXO* regulates relative genitalia size. Both results show that *FOXO* expression negatively affects relative trait size, thus integrating the responses of different tissues and organs to nutritional variation. Small, hornless males generally invest more heavily in testes, ejaculate composition and copulatory organs due to increased sperm competition and the reduced costs of growing horn tissue [42]–[44], [58]. Our results suggest that *FOXO* may play a role in mediating the growth response of genitalia to nutrition and horn growth. In particular, our results suggest that *FOXO* expression may be triggered in the abdomen, which in turn may inhibit the growth of genitalia. This role of *FOXO* in mediating horn-genitalia tradeoffs parallels *FOXO*s role in mediating life history tradeoffs [15]–[17], [34], and is consistent with recent studies demonstrating a role of *FOXO* in organ-specific responses to nutritional variation in *Drosophila* [30].

This work contributes to a growing body of work suggesting insulin signaling as an important mediator of phenotypic plasticity. For instance, the insulin receptor and insulin itself (or insulin-like peptides), show significant differences in expression between polyphenic morphs in a range of systems [29], [33]–[37]. This study is the first to show that *FOXO*, a key growth inhibitor within the insulin pathway, additionally plays a role in the regulation of polyphenic development.

The results of this work suggest several promising areas of future research. A key question for future studies is to investigate whether *FOXO* expression in one body region is directly triggered by the growth of tissue in another body region (such as horns) or alternatively, is regulated by overall body size. Either scenario suggests different mechanisms by which *FOXO* results in trait integration, which could be addressed experimentally by manipulating horn size (via ablation) and nutrition (nutrient supplement or starvation) independent of body size. An additional interesting line of inquiry would be to integrate insulin signaling with endocrine signaling, a known mediator of polyphenisms [59], [60]. Given that both ecdysone and juvenile hormone interact with insulin signaling in insects (e.g., [61], [62]), this would be a promising future approach to better understand and integrate the mechanism of insulin signaling-mediated growth regulation in polyphenisms. Finally, it would be interesting to examine insulin signaling across species that have diverged in body size and the degree of the male polyphenism [63].

## Methods

### Beetle Husbandry

*Onthophagus nigriventris* were collected from populations in Waimea, Hawaii. No specific permits were required for the described field work (this species is not an agricultural pest and is an introduced species) and we had owner permission for collecting on private land. Beetles were maintained in laboratory colonies using established methods [64]. We collected offspring from females setup in separate low-density breeding containers [65]. We transferred larvae in their first or second larval instar from their maternal brood ball to fresh dung in 12-well cell culture plates [66]. Dung in transfer plates had been processed to be similar to maternally processed dung by pressing out as much water as possible using a cheesecloth and paper towels. Once in the 12-well plates, larvae were monitored daily and their transition to third instar recorded for subsequent aging.

### *FOXO* Cloning and Sequencing

A previous EST library for *Onthophagus taurus* had identified a 704 base pair fragment that matched to *FOXO* ([67]; GenBank accession FG540767.1). We used this sequence to design primers to first clone larger fragments of *FOXO* in *O. taurus* and then to clone *FOXO* in our focal species, *O. nigriventris*. Based on these two sequences, we designed a series of primers (see Table S2) that were able to clone fragments of *FOXO* from several species available for comparison (*O. taurus, nigriventris, binodis, sagittarius*, *hecate*, and *pennsylvanicus*). PCR fragments were cloned into a pSC-A vector with the Strataclone PCR Cloning kit (Stratagene/Agilent; Santa Clara, CA). Clones were sequenced using M13 primers and BigDye PCR reactions (see [68]). Partial sequences of all species considered (N = 6) have been submitted to GenBank (accession HQ605917-23).

### *FOXO* Expression in Untreated Individuals

#### Tissue collection

A previous microarray experiment [46] suggested that *FOXO* was differentially expressed between the thoracic and abdominal epidermis in male *O. nigriventris*. We wished to validate this result using real-time quantitative PCR. In particular, we were interested in the tissue-specific scaling of *FOXO* gene expression with body size, in both males and females. Paralleling the methods of previous microarray analyses, we harvested tissue from first-day pupae of *O. nigriventris*. Tissue was harvested from the prothoracic epidermis, which includes the developing horn and the surrounding prothorax. Tissue was also collected from the dorsal abdominal epidermis, avoiding the small lateral projections common in these pupae. The abdomen sample, while containing mostly epidermal tissue, also likely contained minimal amounts of connected adjacent tissue, including muscle and fat body cells.

Tissue was harvested from 21 males and 19 females. All dissections were performed in 1× RNase-free PBS (Ambion/Applied Biosystems, Austin, TX), under RNase-free conditions: all dissecting tools were treated with RNase-Zap (Ambion/Applied Biosystems, Austin, TX). Immediately after removal, tissue was placed in 350 µl Buffer RLT 1% v/v BME (RNeasy Mini Kit, Qiagen, Valencia, CA). Tissue was ground using a sterile, RNase-free pestle fit to the 1.5 µl microcentrifuge tube (Kontes grinders, Kimble Chase, VWR, West Chester, PA) and immediately frozen in liquid nitrogen before being stored at −70 C until RNA extraction.

#### RNA extraction and reverse transcription

RNA was extracted from individual tissue samples using the RNeasy Mini Kit (Qiagen, Valencia, CA), following standard kit protocols. The optional on-column DNA digest was performed (using the Qiagen RNase-free DNase set) to reduce any genomic DNA contamination during the qPCR analyses. Total RNA was eluted in 25 µl RNase-free water (Qiagen, Valencia, CA), and quantified using a Nanodrop (Thermo Scientific, Franklin, MA). Extracted RNA had an average yield of 7.07 µg, and an average purity of 260/230 = 1.94, 260/280 = 2.14. Total RNA was stored at −70°C until further processing for qPCR analyses.

Total RNA was converted to cDNA using the Quantitect Reverse Transcription Kit (Qiagen; Valencia, CA) and standard kit protocols. Briefly, 250 ng total RNA was incubated with 2 ul gDNA wipeout buffer at 42°C for 2 minutes before being combined with Quantiscript reverse transcriptase, 5× buffer, and primer mix and incubated at 42°C for 15 minutes, followed by 85°C for 3 minutes. cDNA was quantified using a Nanodrop 1000 Spectrophotometer (Thermo Scientific, Franklin, MA) and stored at −20 until the qPCR was performed. cDNA had an average yield of 1022 ng/ul, 260/280 ratio of 1.81 and 260/230 ratio of 2.24.

#### Real-time qPCR

Real-time quantitative PCR (qPCR) was performed using SYBR green PCR kit (Qiagen, Valencia, CA) and standard kit protocols. Prior to running our samples, we executed a minimum of five preliminary qPCRs to test a range of primers, control genes and test conditions. We designed primers for qPCR using Oligoanalyzer and PrimerQuest/Primer3 (Integrated DNA Technologies, Coralville, IA). Primers were tested at two concentrations over a four-fold dilution series of cDNA (1000 ng/ul, 100 ng/ul, 10 ng/ul, 1 ng/ul). Based on the Ct data for these standard curves and the dissociation curves from these runs, we chose one primer pair for *FOXO* (for primer sequences, see Table S2).

We used published microarray data [46] to identify possible control genes for our qPCR analyses. Both actin and GAPDH showed low variability in expression across tissues, sexes, and male sizes (mean (SD) in expression (A) was 9.0 (0.28) and 12.5 (0.31) for GAPDH and actin, respectively). We tested three primer pairs each (at two concentrations) for both actin and GAPDH, across a four-fold dilution series. We chose to focus on actin (as opposed to GAPDH) as a control gene because it showed the most robust amplification across the four-fold dilution series (R^2^ of standard curve generally 0.99) and consistent dissociation curves. Final primers for actin were based off of *O. nigriventris* actin sequences (GenBank accession HQ605924, cloned using primers designed off of the *O. taurus* actin sequence GenBank accession FG541406.1). The appropriateness of actin as a control gene was further verified by microarrays that directly hybridized tissues of male *Onthophagus taurus* of varying size [46]. We observed no differential expression of actin, thus meeting a critical requirement for being able to compare *FOXO* expression across individuals of different sizes.

All samples of a particular tissue from a particular sex were run simultaneously (and analyzed together, e.g., female thorax and male abdomen) over four qPCR runs. Based on previous primer optimizations, we ran 1.2 ul of 10 uM actin primers with 250 ng amount of cDNA while 0.6 ul of 10 uM *FOXO* primers were run with 250 ng of cDNA. Standard curves (over a 4-fold dilution), no-template controls and no-reverse transcription controls were included for each primer pair for each qPCR run. Quantitative real time PCR was performed with a Stratagene MX3000P system (Stratagene/Agilent; Santa Clara, CA); SYBR green and ROX (the reference dye) fluorescent measurements were taken every cycle for 45 cycles. PCR settings were as follows: 95°C for 15 min; 45 cycles of 94°C for 15 second, 57°C for 30 second and 72°C for 30 seconds; followed by a 55°C–95°C dissociation curve. A small subset of samples (3 female abdomen samples) failed to amplify and were dropped from the final analysis.

#### qPCR data analysis

We focused on the crossing threshold (referred to as Ct from here-on), for all qPCR analyses. Because we hypothesized *FOXO* to vary with body size and nutrition, we included body size in all analyses. We controlled for the amount of tissue harvested by using a control housekeeping gene (actin) in all analyses [48]. We used an ANOVA to measure the effects of body size (thorax width) and actin expression (both independent variables) on *FOXO* expression. This represents an extension of the multiple regression methods developed by Yuan et al [69] where we are focused on the effect of an additional variable (body size) which is hypothesized to affect gene expression (similar to other model-based approaches to qPCR data that can control for a range of variables, e.g., [70]).

#### In situ hybridization

*In situ*-hybridizations were performed as described previously [71]. DIG-labeled probes were constructed for sense and antisense strands of the same non-homeodomain *FOXO* fragment cloned for RNAinterference, described below (see primers used for cloning in Table S2). Probes were synthesized using using MEGAscript High Yield Transcription Kits (Ambion/Applied Biosystems, Austin, TX). Hybridizations were performed using sagittal sections of pupae. Unlike larval sections, high quality pupal sections are exceedingly difficult to obtain in pupal *Onthophagus* due to the great disparity in densities across tissue types, and we thus regard *in situs* as supplemental to other data. We identified putative tissue types through comparisons across sections and using DAPI staining.

### *FOXO* RNAi Knockdown

We manipulated *FOXO* expression using RNA interference. While it would be ideal to test the effects of both downregulation and upregulation of *FOXO*, current tools available in *Onthophagus* allow permit the former approach.

#### FOXO probe generation

Based on the sequence data generated for several *Onthophagus* species, we tested two primer pairs that would amplify a 250–400 bp sequence outside of the conserved forkhead domain. We chose a pair that spanned a 380 bp range following the conserved forkhead domain (see Table S2 for primer information).

Double-stranded RNA was produced by first using the RNAi *FOXO* primers to amplify and clone the relevant section of *FOXO* into a vector using the pSC-A vector with a Strataclone PCR Cloning kit (Stratagene/Agilent, Santa Clara, CA, USA). M13 primers (Integrated DNA Technologies, Coralville, IA) were used to amplify the relevant fragment, which was gel-extracted, using the Qiaquick Gel Extraction kit (Qiagen, Valencia, CA). We used the MEGAscript kit (and associated protocol; Ambion/Applied Biosystems, Austin, TX) to synthesize RNA. Briefly, an *in-vitro* transcription reaction was run overnight at 37°C using 1 ug of PCR product, NTP mix, reaction buffer and either the T7 or T3 enzyme mix. Control dsRNA was generated using the same methodology applied to vectors with no inserted PCR product. Template DNA was removed by adding 1 ul Turbo DNase (37°C for 15 minutes); RNA was recovered by precipitating with 30 ul LiCl (and 30 ul water and chilling at −20°C for 4 hours). Concentration of product was verified using a Nanodrop (average = 3.57 ug.ul). Complementary RNA strands were annealed by mixing equal amounts of RNA (from with T7 and T3 reactions), heating to 80°C for 5 minutes and then slowly cooling to room temperature over 4 hours in an insulated beaker. dsRNA was brought to 3 ul with injection buffer (5 mM KCl, 1 mM KPO4 pH 6.9) and stored at −70°C until injection.

#### Experimental procedures

Treatment individuals were injected with either 0.5, 1.0, 1.75, or 2.5 ug of double stranded *FOXO* RNA, suspended in 3 ul of injection buffer. Control individuals were injected with 1.0 ug of double-stranded vector RNA suspended in 3 ul of injection buffer. Beetles were injected between day 6 and 10 of the third instar (mean (SD) = 8.31 (1.47)); variation in time of injection did not affect body size or horn-body size scaling. Injections occurred under RNase-free conditions using a gas-tight 1801 Hamilton syringe with a 32-gauge needle. Larvae were injected in the anterior, dorsal abdomen, just under the cuticle.

We measured several phenotypic traits of larvae, pupae and adults. We used pupal thorax width, pupal mass and adult thorax width as measures of body size. The length of the third ( = last) instar, measured in days, was used as an estimate of development time. The third instar constitutes the dominant feeding stage of onthophagine larvae. We measured thoracic horn length (in pupae and adults) as described in [72]. Genitalia investment of males was estimated by first dissecting out the copulatory organ (aedeagus) and then measuring the length (in lateral view) of the paramere (following description in Supplementary material of [58]). We also measured larval mass in a subset of individuals at three time points prior to injection to ensure that any observed effects of knockdown on size were not due to differences in establishment of treatment groups: no differences between control and treatment animals in average larval mass prior to injection were found (*F_1,39_* = 0.02, *P* = 0.88).

#### Knockdown validation

We validated our RNAi knockdown using real-time quantitative PCR. Dissection and qPCR methods were identical to those described above for untreated individuals. We compared gene expression in the prothoracic epidermis and dorsal abdominal epidermis of 11 control-injected and 9 *FOXO*-knockdown individuals (all 11 individuals were 1 ug dsRNA dosage). We used an ANOVA to test for significant differences between control and knock-down individuals, which allowed us to control for differences in body size among individuals. We also estimated the degree of knockdown (fold change) using the methods of Pfaffl [48] by first size-matching control and treatment individuals, and then accounting for differences in efficiency between the control and treatment genes (e.g., for the abdominal tissue run, *E_actin_* = 0.91; *E_FOXO_* = 1.16).

#### Analyses

We fit a sigmoidal curve based on a 4-parameter Hill equation to male body size-horn scaling data using Sigma Plot 2001. This equation was used to calculate expected pupal or adult horn length for a given body size (thorax width). Residual horn length was calculated as the difference between observed and expected horn length for a given body size. For body size-genitalia scaling relationships we used standard ANOVAs instead of reduced major axis methods (errors in variable statistics) sometimes used in allometries [73]. We chose this method because we were interested in relative differences in slopes rather than absolute slope values. Furthermore, the measurement error associated with body size (the x variable) is negligible as thorax width is a highly repeatable measure, making the use of RMA unnecessary [74].

## Supporting Information

Figure S1 **FOXO protein sequences of five Onthophagus species compared to Tribolium castaneum.** Shown are the first 300 amino acids of the gene. The conserved forkhead domain lies approximately between amino acids 80 and 170 (highlighted with the light dotted line). The gene region that made up the RNAi and in situ probe follows the conserved domain (highlighted in heavy dotted line). Darker grays indicate more conserved amino acids.
(TIF)

Figure S2 **Validation of FOXO RNAi knockdown.** Shown are least square means from an ANOVA that included expression of the control gene (actin) and body size (pupal mass) of control-injected and knockdown individuals harvested as first-day pupae (N = 20). Gene expression was measured in the prothoracic epidermis and the abdominal epidermis.
(TIF)

Table S1 Effect of degree of FOXO knockdown on body size. Shown are results of linear regressions considering the effect of FOXO knockdown (0.5, 1.0, 1.75, or 2.5 ug of dsRNA injected) on body size. In this analysis, control individuals were treated as “0.”
(DOC)

Table S2 Primers. Shown are primers used to clone multiple fragments of FOXO from several species of *Onthophagus*; primers used in qPCR analyses; and primers used to clone a non-homeodomain fragment of FOXO from *O. nigriventris* to be used in RNAi and in situs. Base pair regions are with reference to this original FOXO sequence (GenBank accession FG540767.1; the coding region starts at base pair 366) and actin sequence (GenBank accession FG541406.1). A series of primers (sequences available upon request) were used to clone larger fragments of FOXO in the original species (*taurus*) and the focal species (*nigriventris*) before designing the primers reported below, some of which (in particular, the cloning primers) worked in a range of species to clone out fragments of FOXO.
(DOC)
