# Supplementary material for: Insulin Signaling as a Mechanism Underlying Developmental Plasticity: The Role of FOXO in a Nutritional Polyphenism
Source: PLoS One. 2012 Apr 13;7(4):e34857. doi: 10.1371/journal.pone.0034857 (PMC3325941; doi:10.1371/journal.pone.0034857)
Supplement: Table S1 — Effect of degree of FOXO knockdown on body size. Shown are results of linear regressions considering the effect of FOXO knockdown (0.5, 1.0, 1.75, or 2.5 ug of dsRNA injected) on body size. In this analysis, control individuals were treated as “0.” (DOC) [file pone.0034857.s003.doc]

**Table S1.** ***Effect of degree of FOXO knockdown on body size.*** Shown are results of linear regressions considering the effect of FOXO knockdown (0.5, 1.0, 1.75, or 2.5 ug of dsRNA injected) on body size. In this analysis, control individuals were treated as “0.”

|  | *F* | *P* | B | r^2^ |
| --- | --- | --- | --- | --- |
| Pupal Thorax Width (mm) | *F_1,71_* = 7.90 | 0.006 | 0.115 | 0.10 |
| Pupal Mass (g) | *F_1,72_* = 9.89 | 0.002 | 0.011 | 0.12 |
| Adult Thorax Width (mm) | *F_1,60_* = 6.43 | 0.01 | 0.111 | 0.10 |
