# Supplementary material for: Insulin Signaling as a Mechanism Underlying Developmental Plasticity: The Role of FOXO in a Nutritional Polyphenism
Source: PLoS One. 2012 Apr 13;7(4):e34857. doi: 10.1371/journal.pone.0034857 (PMC3325941; doi:10.1371/journal.pone.0034857)
Supplement: Table S2 — Primers. Shown are primers used to clone multiple fragments of FOXO from several species of Onthophagus; primers used in qPCR analyses; and primers used to clone a non-homeodomain fragment of FOXO from O. nigriventris to be used in RNAi and in situs. Base pair regions are with reference to this original FOXO sequence (GenBank accession FG540767.1; the coding region starts at base pair 366) and actin sequence (GenBank accession FG541406.1). A series of primers (sequences available upon request) were used to clone larger fragments of FOXO in the original species (taurus) and the focal species (nigriventris) before designing the primers reported below, some of which (in particular, the cloning primers) worked in a range of species to clone out fragments of FOXO. (DOC) [file pone.0034857.s004.doc]

**Table S2. Primers.** Shown are primers used to clone multiple fragments of FOXO from several species of *Onthophagus*; primers used in qPCR analyses; and primers used to clone a non-homeodomain fragment of FOXO from *O. nigriventris* to be used in RNAi and in situs. Base pair regions are with reference to this original FOXO sequence (GenBank accession FG540767.1; the coding region starts at base pair 366) and actin sequence (GenBank accession FG541406.1). A series of primers (sequences available upon request) were used to clone larger fragments of FOXO in the original species (*taurus*) and the focal species (*nigriventris*) before designing the primers reported below, some of which (in particular, the cloning primers) worked in a range of species to clone out fragments of FOXO.

| **Primer Purpose** | **Gene Region (bp)** | **Forward (5’ - 3’) Reverse (5’- 3’)** |
| --- | --- | --- |
| Cloning^1,2^ | 162-1046 | CTGTATCAACAATGACTGTGAC GGACTTGATTGATTAAAGCTTCC |
| Cloning^1,2^ | 446-1126 | TGTAACACGTGGCCGTTACC GGTATTGGGGATAATCTTCCGC |
| Cloning^1,2^ | 446-1735 | TGTAACACGTGGCCGTTACC GAGCACCCATCAATTGACCCATCA |
| qPCR:^2^ actin | 471-661 | TCACCACCCACGCTGAAAGAGAAA ATAAAGCTTCTGGGCAACGGAACC |
| qPCR:^2^ FOXO | 674-872 | TGGATGGTTCAGAACGTGCCCTAT ACGCCGCTCTTCTTCTCACAGATT |
| Probe (RNAi and in situs)^1^ | 865-1245 | GGCGTCGATGGAAACGAGTAAA TGTTGCTGTTGGATGCTACC |

^1^ *O. taurus* sequences used to design these primers
^2^ *O. nigriventris* sequences used to design these primers
